# Hybrid organic–inorganic crystal structure of 4-(di­methyl­amino)­pyridinium di­methyl­ammonium tetra­chlorido­lead(II)

**DOI:** 10.1107/S2056989017014062

**Published:** 2017-10-20

**Authors:** Cassidy A. Benson, Gage Bateman, Jordan M. Cox, Jason B. Benedict

**Affiliations:** a 730 Natural Sciences Complex, Buffalo, 14260-3000, USA; b 771 Natural Sciences Complex, Buffalo, 14260-3000, USA

**Keywords:** hybrid material, crystal structure, hydrogen bonding

## Abstract

The title compound, (C_2_H_8_N)(C_7_H_11_N_2_)[PbCl_4_], is a hybrid organic–inorganic material. It crystallizes in the space group *C*2/*c* and contains one half of a mol­ecule of lead chloride, 4-(di­methyl­amino)­pyridinium, and di­methyl­ammonium in the asymmetric unit. The crystal structure exhibits chains of lead chloride capped by 4-(di­methyl­amino)­pyridinium and di­methyl­ammoium by hydrogen bonding. This creates a one-dimensional zipper-like structure down the *a* axis. The crystal structure is examined and compared to a similar structure containing lead chloride and di­methyl­benzene-1,4-diaminium.

## Abstract 2

The crystal structure of this hybrid organic–inorganic material exhibits chains of lead chloride capped by 4-(di­methyl­amino)­pyridinium and di­methyl­ammoium by hydrogen bonding. This creates a one-dimensional zipper-like structure down the *a* axis.

## Chemical context   

Hybrid organic–inorganic materials have been gaining attention due to their inter­esting optical properties and for their applications as semiconductors (Dobrzycki & Woźniak, 2008 ▸). Early materials such as lead halogen perovskites have been identified as having intrinsic white-light emission (Dohner *et al.*, 2014 ▸) and as an inexpensive and high conversion material for solar cells (Baikie *et al.*, 2013 ▸; Zhao & Zhu, 2014 ▸). By changing the size and structure of the organic portions of these materials, one can begin to assess the impact of these groups on the resulting crystal structures (Gillon *et al.*, 2000 ▸). For organic cations containing groups capable of hydrogen bonding, these inter­actions may form the basis for deliberate crystal engineering of next generation materials. Herein we report the structure of a new hybrid organic–inorganic mat­erial that contains 1-D lead chloride chains capped by di­methyl­ammonium (DMA) and 4-(di­methyl­amino)­pyridinium (4DAP) groups through extensive hydrogen-bonding inter­actions.
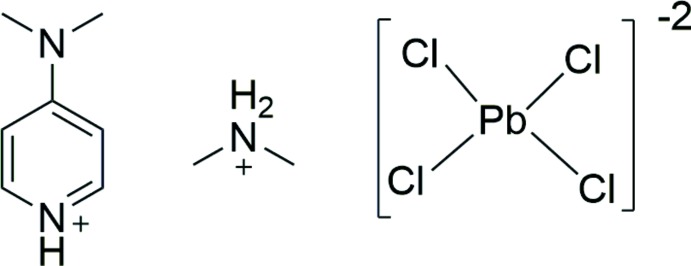


## Structural commentary   

**Figure 1:**
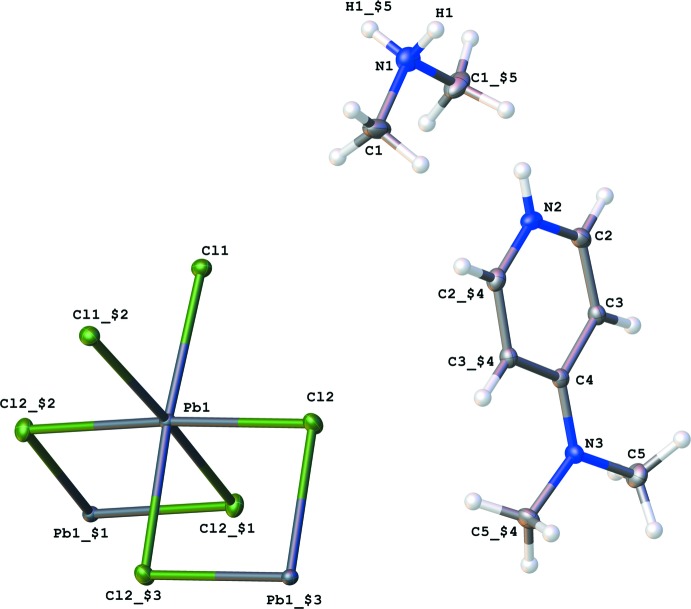
The expanded asymmetric unit of the title crystal structure, showing the naming scheme. The asymmetric unit contains half of each component: di­methyl­ammonium, 4-(di­methyl­amino)­pyridinium, and [PbCl_4_]^2−^. Displace­ment ellipsoids are drawn at the 50% probability level. Atom colors: carbon (gray), nitro­gen (blue), hydrogen (white), lead (dark blue) and chlorine (green). [Symmetry operators: ($1) *x*, 1 − *y*, −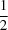 + *z*; ($2) 2 − *x*, *y*, 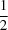 − *z*; ($3) 2 − *x*, 1 − *y*, 1 − *z*; ($4) 1 − *x*, *y*, 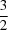 − *z*; ($5) 1 − *x*, *y*, 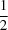 − *z*.]

This structure crystallizes in the centrosymmetric space group *C*2/*c* with half of a mol­ecule of DMA, half of a [PbCl_4_]^2−^anion, and half of a mol­ecule of 4DAP in the asymmetric unit, shown in Fig. 1 ▸. The point groups of the two cations are *C*_2_. The lead metal center, with its six chloride ligands, exhibits a slightly distorted octa­hedral coordination geometry (formally *C*_2_ symmetry). Of the two crystallographically unique Cl atoms, Cl1 and its symmetry equivalent produced through a *C*_2_ rotation are the two terminal atoms [Pb—Cl = 2.8499 (4) Å]. The other crystallographically unique Cl atom, Cl2, produces the four bridging atoms through a *C*_2_ rotation and inversion operation that results in an additional two unique Pb—Cl bonds [2.9015 (5) and 2.9041 (5) Å]. These bridging ligands form one-dimensional chains of [PbCl_4_]^2−^ anions which extend approximately along the [001] direction. The net negative two charge of the lead chloride anion is balanced by the positive charges of the DMA and 4DAP cations.

## Supra­molecular features   

**Figure 2:**
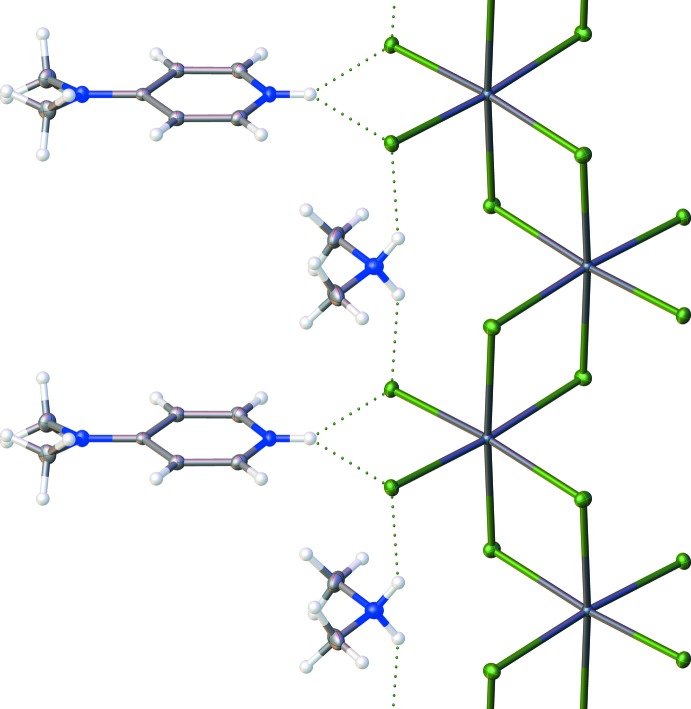
Hydrogen-bonding network in a single PbCl_4_ chain viewed down [100]. Displacement ellipsoids are drawn at the 50% probability level. Atom colors: carbon (gray), nitro­gen (blue), hydrogen (white), lead (dark blue) and chlorine (green).

**Figure 3:**
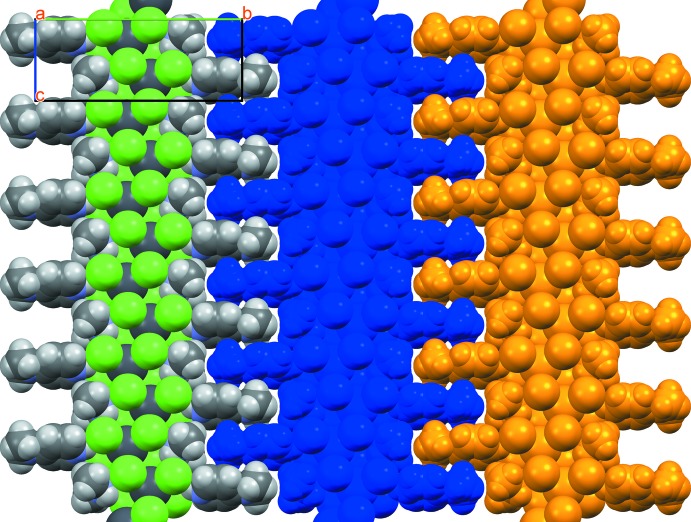
Space-filling model of inter­calated PbCl_4_ chains viewed down [100]. (Left) PbCl_4_ chain colored by element. (Center and right) Intercalation illustrated by chains coloured in blue and orange..

The one-dimensional lead chloride chains are capped in the [010] direction by 4DAP and DMA mol­ecules *via* hydrogen bonds (Table 1 ▸), which form between the NH groups on the 4DAP and DMA mol­ecules and the terminal chloride ligands on each lead atom. The hydrogen-bond donor on the 4DAP forms a hydrogen bond with each of the two terminal chlorides on the nearest Pb atom, while each donor on the DMA forms a hydrogen bond with one terminal chloride on Pb atoms that are separated in the chain by one additional Pb. The hydrogen-bonding network of the title structure is shown in Fig. 2 ▸. The chains are packed together along the [010] direction by inter­calation of the peripheral 4DAP ligands, as shown in Fig. 3 ▸, to form sheets which lie in the (100) plane. These sheets are held together primarily by weak inter­molecular inter­actions, although one CH⋯Cl contact (2.788 Å) does exist that is 0.162 Å less than the sum of the van der Waals radii for these atoms.

## Database survey   

**Figure 4:**
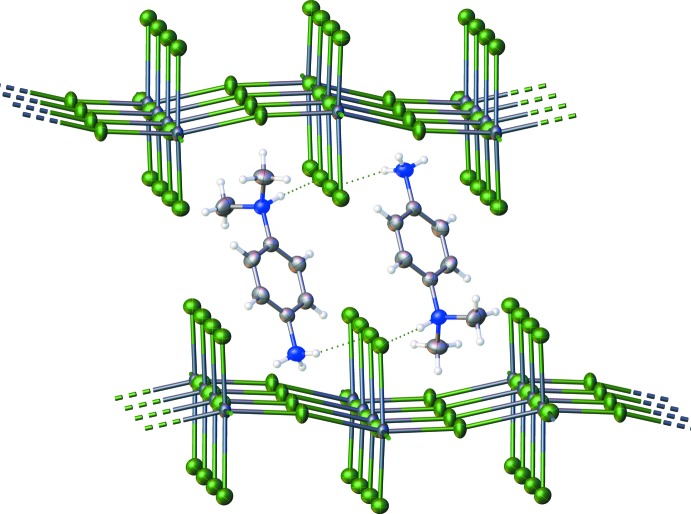
Hydrogen-bonding network in [PbCl_4_]^2−^·di­methyl­benzene-1,4-diam­in­ium. Displacement ellipsoids are drawn at the 50% probability level. Atom colors: carbon (gray), nitro­gen (blue), hydrogen (white), lead (dark blue) and chlorine (green).

The crystal structure of [PbCl_4_]^2−^ and di­methyl­benzene-1,4 diaminium was reported in 2008 (Dobrzycki & Woźniak, 2008 ▸). This compound crystallizes in the centrosymmetric space group *P*2_1_/*n*, and the structure contains two-dimensional lead chloride sheets that run parallel to [001]. Like in the title structure, each Pb center possesses approximately octa­hedral symmetry (formally *C*_1_) and is coordinated to two terminal and four bridging chloride atoms. Two of the bridging atoms are crystallographically unique and each give rise to a symmetry-related atom to yield four bonds to the Pb center (Pb—Cl2 = 2.945 and 2.927 Å; Pb—Cl3 = 3.095 and 2.765 Å). In this compound, hydrogen bonding occurs between the terminal chloride ligands and the protons on the diaminium groups of the organic cation, as shown in Fig. 4 ▸. Both structures are similarly charge-balanced in that the respective anionic PbCl_4_ sheet or chain is balanced by the positive charge from organic cation mol­ecules. The major difference between these two compounds is that the title structure contains one-dimensional chains while this structure contains two-dimensional sheets.

## Synthesis and crystallization   

To a 20 mL scintillation vial was added 4DAP (1.008 g, 8.25 × 10 ^−3^ mol) and concentrated hydro­chloric acid (2 ml) creating an acidic solution. To a 23 mL screw-top thick-walled vial was added PbCl_2_ (0.5040 g, 1.81 × 10 ^−3^ mol), DMF (1 ml), and the 4DAP acid solution (1 ml). The thick-walled vial was placed in the oven for 11 days at 373 K. Clear, colorless crystals of the title compound that were suitable for single-crystal X-ray diffraction were obtained. The DMA is present in the lattice due to the *in situ* degradation of DMF, which can occur at the reaction temperature (Burrows *et al.*, 2008 ▸).

## Refinement   

Crystal data, data collection and structure refinement details are summarized in Table 2 ▸. The position of the ammonium hydrogen atom was determined from the difference-Fourier map, and all other hydrogen atoms were placed in idealized positions with bond lengths set to 0.98 Å for alkyl C—H protons, 0.95 Å for aliphatic C—H protons, and 0.88 Å for the pyridinium proton. These hydrogen atoms were refined using a riding model with *U*_iso_(H) = 1.2 *U*_eq_(N,C) for all N—H and aliphatic protons and 1.5 *U*_eq_(C) for methyl group protons. To appropriately model the ammonium hydrogen atom, which is complicated by the site of symmetry on which the nitro­gen atom resides, the distance between the hydrogen atom and its symmetry-equivalent was restrained to 1.4 (2) Å. No other constraints were applied to the refinement model.

## Supplementary Material

Crystal structure: contains datablock(s) I. DOI: 10.1107/S2056989017014062/nr2065sup1.cif

Structure factors: contains datablock(s) I. DOI: 10.1107/S2056989017014062/nr2065Isup2.hkl

Supporting information file. DOI: 10.1107/S2056989017014062/nr2065Isup3.mol

CCDC reference: 1577164

Additional supporting information:  crystallographic information; 3D view; checkCIF report

## Figures and Tables

**Table 1:** Hydrogen-bond geometry (Å, °)

*D*—H⋯*A*	*D*—H	H⋯*A*	*D*⋯*A*	*D*—H⋯*A*
N1—H1⋯Cl1^i^	0.87 (2)	2.47 (1)	3.1844 (6)	139 (2)
N2—H2⋯Cl1^i^	0.88	2.63	3.2901 (18)	133
N2—H2⋯Cl1^ii^	0.88	2.63	3.2901 (18)	133

Symmetry codes: (i) 
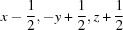
; (ii) 
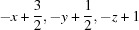
.

**Table 2:** Experimental details

Crystal data	
Chemical formula	(C_2_H_8_N)(C_7_H_11_N_2_)[PbCl_4_]
*M*_r_	518.26
Crystal system, space group	Monoclinic, *C*2/*c*
Temperature (K)	90
*a*, *b*, *c* (Å)	11.0965 (11), 19.120 (2), 7.5453 (8)
β (°)	91.0813 (19)
*V* (Å^3^)	1600.6 (3)
*Z*	4
Radiation type	Mo *K*α
μ (mm^−1^)	11.19
Crystal size (mm)	0.12 × 0.10 × 0.04

Data collection	
Diffractometer	Bruker SMART APEXII area detector
Absorption correction	Multi-scan (*SADABS*; Bruker, 2016 ▸)
*T*_min_, *T*_max_	0.291, 0.746
No. of measured, independent and observed [*I* > 2σ(*I*)] reflections	14318, 2461, 2398
*R*_int_	0.030
(sin θ/λ)_max_ (Å^−1^)	0.716

Refinement	
*R*[*F* ^2^ > 2σ(*F* ^2^)], *wR*(*F* ^2^), *S*	0.013, 0.034, 1.04
No. of reflections	2461
No. of parameters	85
No. of restraints	1
H-atom treatment	H atoms treated by a mixture of independent and constrained refinement
Δρ_max_, Δρ_min_ (e Å^−3^)	1.68, −0.76

Computer programs: *APEX2* and *SAINT* (Bruker, 2016 ▸), *olex2.solve* (Bourhis *et al.*, 2015 ▸), *SHELXL2016* (Sheldrick, 2015 ▸) and *OLEX2* (Dolomanov *et al.*, 2009 ▸).

## supplementary crystallographic information

### Crystal data

**Table:**

(C_2_H_8_N)(C_7_H_11_N_2_)[PbCl_4_]	*F*(000) = 976
*M**_r_* = 518.26	*D*_x_ = 2.151 Mg m^−^^3^
Monoclinic, *C*2/*c*	Mo *K*α radiation, λ = 0.71073 Å
*a* = 11.0965 (11) Å	Cell parameters from 9977 reflections
*b* = 19.120 (2) Å	θ = 3.4–30.6°
*c* = 7.5453 (8) Å	µ = 11.19 mm^−^^1^
β = 91.0813 (19)°	*T* = 90 K
*V* = 1600.6 (3) Å^3^	Plate, colourless
*Z* = 4	0.12 × 0.10 × 0.04 mm

### Data collection

**Table:**

Bruker SMART APEXII area detector diffractometer	2461 independent reflections
Radiation source: microfocus rotating anode, Incoatec Iµs	2398 reflections with *I* > 2σ(*I*)
Mirror optics monochromator	*R*_int_ = 0.030
Detector resolution: 7.9 pixels mm^-1^	θ_max_ = 30.6°, θ_min_ = 2.1°
ω and φ scans	*h* = −15→15
Absorption correction: multi-scan (SADABS; Bruker, 2016)	*k* = −27→27
*T*_min_ = 0.291, *T*_max_ = 0.746	*l* = −10→10
14318 measured reflections	

### Refinement

**Table:**

Refinement on *F*^2^	1 restraint
Least-squares matrix: full	Hydrogen site location: mixed
*R*[*F*^2^ > 2σ(*F*^2^)] = 0.013	H atoms treated by a mixture of independent and constrained refinement
*wR*(*F*^2^) = 0.034	*w* = 1/[σ^2^(*F*_o_^2^) + (0.0197*P*)^2^ + 0.6272*P*] where *P* = (*F*_o_^2^ + 2*F*_c_^2^)/3
*S* = 1.04	(Δ/σ)_max_ = 0.001
2461 reflections	Δρ_max_ = 1.68 e Å^−^^3^
85 parameters	Δρ_min_ = −0.76 e Å^−^^3^

### Special details

**Table:**

Geometry. All esds (except the esd in the dihedral angle between two l.s. planes) are estimated using the full covariance matrix. The cell esds are taken into account individually in the estimation of esds in distances, angles and torsion angles; correlations between esds in cell parameters are only used when they are defined by crystal symmetry. An approximate (isotropic) treatment of cell esds is used for estimating esds involving l.s. planes.

### Fractional atomic coordinates and isotropic or equivalent isotropic displacement parameters (Å^2^)

**Table:**

	*x*	*y*	*z*	*U*_iso_*/*U*_eq_	
Pb1	1.000000	0.44266 (2)	0.250000	0.00845 (3)	
Cl2	0.86249 (4)	0.44764 (2)	0.57303 (6)	0.01412 (8)	
Cl1	0.85512 (3)	0.33250 (2)	0.10684 (5)	0.01280 (7)	
N1	0.500000	0.18730 (13)	0.250000	0.0181 (4)	
H1	0.4713 (19)	0.1598 (13)	0.3311 (18)	0.022*	
N3	0.500000	0.52247 (11)	0.750000	0.0121 (4)	
N2	0.500000	0.30724 (11)	0.750000	0.0138 (4)	
H2	0.499998	0.261213	0.750000	0.017*	
C4	0.500000	0.45287 (12)	0.750000	0.0103 (4)	
C2	0.40384 (16)	0.34254 (9)	0.6811 (2)	0.0138 (3)	
H2A	0.337557	0.317062	0.632503	0.017*	
C3	0.40049 (14)	0.41371 (9)	0.6802 (2)	0.0121 (3)	
H3	0.331759	0.437346	0.633026	0.015*	
C5	0.39118 (18)	0.56252 (9)	0.7039 (3)	0.0165 (4)	
H5A	0.320144	0.537848	0.747178	0.025*	
H5B	0.384567	0.567587	0.574804	0.025*	
H5C	0.396086	0.608909	0.758791	0.025*	
C1	0.5973 (2)	0.23118 (11)	0.3336 (3)	0.0238 (4)	
H1A	0.641230	0.255783	0.240978	0.036*	
H1B	0.653055	0.201124	0.400987	0.036*	
H1C	0.561169	0.265369	0.413504	0.036*	

### Atomic displacement parameters (Å^2^)

**Table:**

	*U*^11^	*U*^22^	*U*^33^	*U*^12^	*U*^13^	*U*^23^
Pb1	0.00920 (5)	0.00796 (5)	0.00818 (5)	0.000	0.00013 (3)	0.000
Cl2	0.01194 (18)	0.01588 (19)	0.01458 (19)	−0.00345 (13)	0.00121 (15)	−0.00144 (13)
Cl1	0.01214 (16)	0.01147 (16)	0.01476 (17)	−0.00031 (13)	−0.00059 (13)	−0.00139 (13)
N1	0.0206 (11)	0.0187 (11)	0.0151 (10)	0.000	0.0012 (8)	0.000
N3	0.0089 (8)	0.0123 (9)	0.0149 (9)	0.000	−0.0014 (7)	0.000
N2	0.0176 (10)	0.0103 (9)	0.0136 (9)	0.000	0.0019 (7)	0.000
C4	0.0102 (10)	0.0123 (10)	0.0085 (10)	0.000	0.0009 (8)	0.000
C2	0.0132 (7)	0.0152 (8)	0.0131 (7)	−0.0030 (6)	0.0011 (6)	−0.0018 (6)
C3	0.0098 (6)	0.0143 (7)	0.0121 (7)	−0.0003 (6)	0.0002 (5)	−0.0003 (6)
C5	0.0131 (8)	0.0165 (8)	0.0200 (9)	0.0044 (6)	−0.0016 (7)	0.0015 (6)
C1	0.0315 (10)	0.0156 (8)	0.0247 (9)	−0.0080 (7)	0.0084 (8)	−0.0049 (7)

### Geometric parameters (Å, º)

**Table:**

Pb1—Cl2	2.9015 (5)	N2—C2	1.358 (2)
Pb1—Cl2^i^	2.9041 (5)	N2—C2^v^	1.358 (2)
Pb1—Cl2^ii^	2.9015 (5)	C4—C3^v^	1.427 (2)
Pb1—Cl2^iii^	2.9041 (5)	C4—C3	1.427 (2)
Pb1—Cl1^ii^	2.8500 (4)	C2—H2A	0.9500
Pb1—Cl1	2.8499 (4)	C2—C3	1.361 (2)
N1—H1^iv^	0.872 (17)	C3—H3	0.9500
N1—H1	0.872 (17)	C5—H5A	0.9800
N1—C1^iv^	1.497 (2)	C5—H5B	0.9800
N1—C1	1.497 (2)	C5—H5C	0.9800
N3—C4	1.331 (3)	C1—H1A	0.9800
N3—C5^v^	1.466 (2)	C1—H1B	0.9800
N3—C5	1.466 (2)	C1—H1C	0.9800
N2—H2	0.8800		
			
Cl2—Pb1—Cl2^ii^	176.237 (16)	C2—N2—H2	119.8
Cl2^ii^—Pb1—Cl2^iii^	94.728 (14)	C2^v^—N2—H2	119.8
Cl2—Pb1—Cl2^iii^	82.538 (14)	C2^v^—N2—C2	120.4 (2)
Cl2—Pb1—Cl2^i^	94.728 (14)	N3—C4—C3^v^	121.65 (10)
Cl2^iii^—Pb1—Cl2^i^	87.519 (19)	N3—C4—C3	121.66 (10)
Cl2^ii^—Pb1—Cl2^i^	82.539 (14)	C3^v^—C4—C3	116.7 (2)
Cl1—Pb1—Cl2^ii^	90.441 (13)	N2—C2—H2A	119.3
Cl1^ii^—Pb1—Cl2	90.442 (13)	N2—C2—C3	121.33 (16)
Cl1—Pb1—Cl2^i^	94.115 (15)	C3—C2—H2A	119.3
Cl1^ii^—Pb1—Cl2^ii^	92.339 (12)	C4—C3—H3	119.9
Cl1—Pb1—Cl2	92.340 (13)	C2—C3—C4	120.13 (16)
Cl1—Pb1—Cl2^iii^	174.742 (12)	C2—C3—H3	119.9
Cl1^ii^—Pb1—Cl2^iii^	94.116 (15)	N3—C5—H5A	109.5
Cl1^ii^—Pb1—Cl2^i^	174.741 (12)	N3—C5—H5B	109.5
Cl1—Pb1—Cl1^ii^	84.698 (18)	N3—C5—H5C	109.5
Pb1—Cl2—Pb1^iii^	97.462 (14)	H5A—C5—H5B	109.5
H1—N1—H1^iv^	106 (3)	H5A—C5—H5C	109.5
C1^iv^—N1—H1^iv^	108.2 (15)	H5B—C5—H5C	109.5
C1—N1—H1^iv^	111.3 (15)	N1—C1—H1A	109.5
C1^iv^—N1—H1	111.3 (15)	N1—C1—H1B	109.5
C1—N1—H1	108.2 (15)	N1—C1—H1C	109.5
C1^iv^—N1—C1	111.8 (2)	H1A—C1—H1B	109.5
C4—N3—C5	121.49 (10)	H1A—C1—H1C	109.5
C4—N3—C5^v^	121.49 (10)	H1B—C1—H1C	109.5
C5^v^—N3—C5	117.0 (2)		
			
N3—C4—C3—C2	179.51 (11)	C5—N3—C4—C3^v^	−170.46 (13)
N2—C2—C3—C4	1.0 (2)	C5^v^—N3—C4—C3^v^	9.54 (13)
C2^v^—N2—C2—C3	−0.51 (12)	C5^v^—N3—C4—C3	−170.46 (13)
C3^v^—C4—C3—C2	−0.49 (11)	C5—N3—C4—C3	9.54 (13)

Symmetry codes: (i) *x*, −*y*+1, *z*−1/2; (ii) −*x*+2, *y*, −*z*+1/2; (iii) −*x*+2, −*y*+1, −*z*+1; (iv) −*x*+1, *y*, −*z*+1/2; (v) −*x*+1, *y*, −*z*+3/2.

### Hydrogen-bond geometry (Å, º)

**Table:**

*D*—H···*A*	*D*—H	H···*A*	*D*···*A*	*D*—H···*A*
N1—H1···Cl1^vi^	0.87 (2)	2.47 (1)	3.1844 (6)	139 (2)
N2—H2···Cl1^vi^	0.88	2.63	3.2901 (18)	133
N2—H2···Cl1^vii^	0.88	2.63	3.2901 (18)	133

Symmetry codes: (vi) *x*−1/2, −*y*+1/2, *z*+1/2; (vii) −*x*+3/2, −*y*+1/2, −*z*+1.
